# Relative bioavailability of L-methionine and DL-methionine in growing broilers

**DOI:** 10.1016/j.psj.2024.104311

**Published:** 2024-09-07

**Authors:** Elham Izadi, Mohammad Hossein Shahir, Mohammad Amir Karimi Torshizi

**Affiliations:** ⁎ Department of Animal Science, Faculty of Agriculture, University of Zanjan, P.O. Box: 45371-38791, Zanjan, Iran; † Department of Poultry Science, Faculty of Agriculture, Tarbiat Modares University, Tehran, Iran

**Keywords:** methionine source, bioavailability, blood metabolite, gut morphology, broiler

## Abstract

Two separate studies were conducted in growing broiler chickens to examine the relative bioavailability (**RBA**) of L-smethionine (**L-Met**) vs. DL-methionine (**DL-Met**) in the starter (0–10 d, Experiment 1) and grower (11–24 d, Experiment 2) periods. In each experiment, 540 male Arian broilers were weighed and randomly allocated to nine dietary treatments in a completely randomized design with 6 replicates: basal diet (**BD**) with no methionine (**Met**) supplementation and eight diets supplemented with incremental levels (0.8, 1.6, 2.4, and 3.2 g/kg) of DL-methionine (**DL-Met**) or L-methionine (**L-Met**). Supplementation of the BD with either DL-Met or L-Met improved growth performance (*P* < 0.05), breast percentage (*P* < 0.05), and antioxidant status (*P* < 0.05) of broilers in both experiments. Orthogonal contrasts showed that L-Met supplementation compared to DL-Met (specifically at levels 0.8 and 1.6 g/kg) improved average daily gain (**ADG**, *P* < 0.05), average daily feed intake (**ADFI**, *P* < 0.01), and feed to gain ratio (**F:G**, *P* < 0.01) in the starter phase. In the grower phase, L-Met supplementation (specifically at levels 0.8 and 1.6 g/kg) only improved F:G (*P* < 0.05) compared to DL-Met, with no significant differences in the other performance parameters. Nonlinear regression analysis showed that RBA of L-Met based on carcass percentage was significantly (*P* < 0.05) higher than that of DL-Met in the starter phase. Based on the findings of this study, it seems that using L-Met compared to DL-Met may improve the feed efficiency and carcass percentage of young growing broiler chickens.

## INTRODUCTION

Methionine (**Met**) is well known as the first limiting essential amino acid for broiler chickens in corn and soybean meal-based diets (Baker, 2006; Dilger and Baker, 2007). It is a critical amino acid for the initiation of protein synthesis. It participates in many important biological functions within the body, such as trans-methylation and trans-sulfuration pathways for the synthesis of sulfur-containing bioactive compounds, such as glutathione and taurine (Baker, 2005; Bunchasak, 2009). Methionine undersupply affects most biological processes, such as the cellular antioxidant system (Mukwevho et al., 2014; Shen et al., 2015; Park et al., 2018), leading to a significant reduction in performance and feed efficiency (Elwinger et al., 2008). Therefore, it is extremely important to supplement this critical amino acid (Dilger and Baker, 2007).

Synthetic Met products such as L-Met and DL-Met (racemic mixture of D-Met and L-Met) are normally used for the fortification of poultry feed. DL-Met has been conventionally added to poultry feeds for many years; however, L-Met has recently become commercially available (Wang et al., 2019). Dietary D-Met must be converted to L-Met by enzymatic reactions within the body for the incorporation into body proteins. It has generally been accepted that dietary D-Met can be converted to L-Met with high efficiency because the activity of D-amino acid oxidase in converting D-Met to L-Met has been detected in the liver, kidney, and small intestine of chicks (Zhang et al., 2019). However, the expression of D-amino acid oxidase is significantly low in young animals (D'Aniello et al., 1993). L-Met is the only biologically functional form of Met readily utilized by intestinal cells in young animals; therefore, compared with DL-Met, L-Met may have a better effect on the growth performance of young broiler chicks (Shen et al., 2015). Previous studies have suggested that L-Met and DL-Met have the same efficacy in growing chicks (Dilger and Baker, 2007; Dilger et al., 2007; Zhang et al., 2017) and pigs (Kong et al., 2016). A few studies have recently questioned this assumption by comparing the biological efficacy of Met sources and acknowledged that L-Met is superior to DL-Met in broiler chicks (Shen et al., 2015; Esteve-Garcia and Khan, 2018; Wang et al., 2019), turkey poults (Park et al., 2018), and ducklings (Zhang et al., 2019).

Due to inconsistencies in the efficacy of these 2 products, further research is needed to determine the bioavailability of L-Met relative to DL-Met, which is the most commonly used Met source. To the best of our knowledge, there has been no separate comparative research on the bioavailability of L-Met relative to DL-Met in starting and growing broiler chicken. The objective of the present experiment was to separately survey the relative bioavailability of L-Met compared to conventional DL-Met in starting and growing broilers based on performance, carcass traits, antioxidant status, and intestinal villus morphology criteria.

## MATERIALS AND METHODS

The Animal Care and Use Committee of University of Zanjan, Iran approved experimental protocols using chicken in this work (Approval No. 13961102).

### Birds Management

Two separate experiments (**Exp**) were carried out in the starter (Exp. 1; 0–10 d) and grower (Exp. 2; 11–24 d) phases. In each experiment, different batches of birds were used. A total of 1080 male Arian broiler chicks (540 chicks for each experiment) were acquired from a commercial hatchery (Arian strain, Babol-Kenar Line Breeding Center) and assigned to 54 experimental pens (10 chicks per pen; 1 m^2^) that contained two nipple drinkers and a feeder. All chickens underwent spray vaccination for infectious bronchitis and Newcastle disease on d 1 (on the experimental farm) and d 19 of life, and Gumboro vaccination was administered on d 13. Feed and water were provided ad libitum. All chicks in the Exp. 2 were fed a standard diet with equal portions of both Met sources until 11 d of age and then weighed and randomly assigned to the experimental pens. Arian's broiler management guideline was used for brooding and lighting during the experiments. Briefly, the temperature was set at 32°C at chick placement and then gradually declined by 0.5°C every day to a final set point of 24°C at 22 d of age.

### Diet Formulation and Experimental Design

Two basal diets were formulated without Met supplementation for both experiments (Table 1). The BD for the starter (Exp. 1) and grower (Exp. 2) phases contained 2.9 and 2.7 g/kg standardized ileal digestible (**SID**) Met, respectively, which represented approximately 60% of the Met requirement of broilers (Ministry of Agricultre Jihad, 2020). The basal diets were divided into nine parts and were supplemented with graded levels (0, 0.8, 1.6, 2.4, and 3.2 g/kg) of DL-Met (Evonik Industries AG, Hanau, Germany) or L-Met (CJ Cheil Jedang Co., Seoul, South Korea) which brought dietary SID TSAA content to 60, 70, 80, 90, and 100% of the requirement (Ministry of Agricultre Jihad, 2020). Both DL-Met and L-Met crystals had 99% purity. The prepared mash diets were then pelleted using a steam pellet mill (FDSP SZLH32 model, Jiangsu, China). The conditioning time was approximately 10 s at 70°C and pressure of 1.2 kg/cm^2^. Five kilograms from the middle part of each batch of pelleted feeds were collected and kept in nylon bags for physical quality and chemical tests. The pellet durability indexes of the experimental diets were estimated to be 84 to 86% and 85 to 88% for the starter and grower phases, respectively.

**Table 1:** Ingredients and chemical composition of the basal diets (g/kg, as-fed basis).

Ingredients (g/kg)	Starter (0–10 d.)	Grower (11–24 d.)
Corn	525	570
Soybean meal	395	345
Soybean oil	26	33
DL-Met	0	0
L-Met	0	0
L-Lys. HCl	1.45	1.2
L-Threonine	1.10	1.1
Di-calcium phosphate	20.50	20
Calcium carbonate	13.50	11.5
Sodium bicarbonate	1.60	1.6
Sodium chloride	2.40	2.4
Vitamin premix ^1^	2.50	2.5
Mineral premix^2^	2.50	2.5
Bentonite	8.45	9.2
Calculated nutrients		
Metabolizable energy (MJ/kg)	12.14	12.55
Crude protein	222	202
Calcium	10.3	9.3
Available phosphorus	4.5	4.4
Dietary amino acid contents (g/kg)		
Digestible lysine (SID)^3^	12.4	11.3
Digestible Methionine (SID)	2.9	2.7
Digestible Cystine (SID)	3	2.7
Digestible Met + Cystine (SID)	5.9	5.4
Digestible Threonine (SID)	8.3	7.5
Digestible Tryptophan (SID)	2.4	2.1
Digestible Arginine (SID)	12.9	12.2
Digestible Isoleucine (SID)	8.7	8.3
Digestible Leucine (SID)	15.6	14.9
Digestible Valine (SID)	9.6	9
Digestible Glutamic acid (SID)	59	46
Digestible Phenylalanine (SID)	11.1	8.9
Digestible Histidine (SID)	5	4.1
Digestible Aspartic acid (SID)	17.5	14.8
Digestible Glycine (SID)	7.8	7.6
Digestible Alanine (SID)	9	8.7
Digestible Proline (SID)	11.5	9.1
Digestible Serine (SID)	9.5	8.4
Digestible Tyrosine (SID)	6.8	5.7
Analyzed nutrients		
Crude protein	217	199
Methionine (total)	3.2	2.9

^1^Vitamin premix provided per kilogram of diet: retinol, 11,000 IU; cholecalciferol, 5,000 IU; tocopheryl acetate, 55 mg; vitamin K_3_, 2.2 mg; vitamin B_1_, 2.2 mg; vitamin B_2_, 5.4 mg; vitamin B_6_, 3 mg; vitamin B_12_, 16. mg; niacin, 55 mg; pantothenic acid, 15 mg; folic acid, 1.8 mg and choline, 1600 mg.

2 Mineral premix provided per kilogram of diet: Mn as manganese oxide, 120 mg; Zn as zinc sulfate, 110 mg; Fe as ferrous sulfate, 20 mg; Cu as copper sulfate, 16 mg; Selenium as sodium Selenite 0.2 mg.

3 Standardized ileal digestible

Nine dietary treatments with six replicates were arranged in a completely randomized design. The control group birds were fed BD without supplemental Met, which contained 3.2 and 2.9 g/kg total Met for Exp. 1 and 2, respectively whereas the treatment group birds received diets supplemented with four graded levels (0.8, 1.6, 2.4, and 3.2 g/kg) of DL-Met and four graded levels (0.8, 1.6, 2.4, and 3.2 g/kg) of L-Met. High-performance liquid chromatography (Waters, Model: 2695E, USA) was used to determine the amino acid content of diets following hydrolysis by hydrochloric acid (6 N) and derivatization by orthophaldialdehyde (OPA) (Table 1, Table 2). Standardized ileal digestibility (SID) values for all amino acids were calculated using the AminoDat5 software (Evonik, Germany).

**Table 2:** The analyzed values for supplemented methionine plus cystine (total) in diets.

Treatments	Met source	Supplemental level (g/kg)	Starter (0–10 d.) (g/kg)	Grower (11–24 d.) (g/kg)
1	BD	0	6.6	6.2
2	DL-Met	0.8	7.5	7
3	DL-Met	1.6	8.3	7.9
4	DL-Met	2.4	9.2	8.7
5	DL-Met	3.2	1.05	9.6
6	L-Met	0.8	7.6	7
7	L-Met	1.6	8.3	8
8	L-Met	2.4	9.3	8.7
9	L-Met	3.2	1	9.7

### Growth Performance

The chicks were weighed at the beginning of each experiment and randomly assigned to the experimental pens. Body weight (**BW**) and feed intake (**FI**) were recorded at the end of each period, and mortality was monitored daily. Average daily gain (ADG), average daily feed intake (ADFI), and feed-to-gain ratio (F:G) were calculated and adjusted for any mortality.

### Biochemical Assay of Serum

At the end of each experiment (10 and 24 d of age), two birds close to the average weight of each pen were selected for blood collection using heparin tubes. Blood samples were centrifuged at 3,000 × *g* for 10 min at 4°C, and the obtained sera were stored at - 20°C for laboratory analysis. Serum concentrations of total protein, albumin, and uric acid were measured using an auto-analyzer (Cobas 6,000; Roche diagnostics, Basel, Switzerland) with the respective diagnostic kits. Serum globulin concentrations were calculated by subtracting albumin concentrations from total protein concentration. Malondialdehyde (**MDA**) concentration, as an index of lipid peroxidation, was assayed using the thiobarbituric acid colorimetric method (Nair and Turner, 1984). Glutathione peroxidase (**GSH-Px_2_**) activity was determined using a commercial kit (RANDOX Laboratories Ltd., London, UK).

### Carcass Traits

At the end of each experiment, two birds close to the average weight of each pen were weighed and euthanized by cervical dislocation after deprivation for 6 h. After blood and feathers were removed, hot carcasses were weighed, and head, feet, abdominal fat pad, and all of the viscera, except the lungs and kidneys, were further removed to determine carcass yield based on live weight. Breast muscle, abdominal fat, and liver were excised, weighted, and expressed as a percentage of the live weight.

### Intestinal Histology Measurement

The small intestine was divided into three segments: the duodenum (from the gizzard outlet to the end of the pancreatic loop), the jejunum (from the end of the pancreatic loop to Meckel's diverticulum), and the ileum (from Meckel's diverticulum to the ileocecal junction). The duodenum, jejunum, and ileum contents were emptied by gentle washing with buffer saline, and their length and weight were measured. To survey intestinal morphology, jejunum segments were fixed with 10% formalin solution and prepared using the standard paraffin embedding procedure (Shen et al., 2009) by sectioning at a thickness of 5 μm and staining with hematoxylin and eosin. Subsequently, the segments were examined under a Sony CCD color video camera attached to an Olympus Van-Ox S microscope (Opelco, Washington, DC). Morphological indicators, such as villus height (from the tip of the villus to the villus-crypt junction), villus width (width of the villus at one-half of the villus height), and crypt depth (from the junction to the base of the crypt) were determined. Ten well-oriented intact villi and their associated crypts were measured in each slide. Analyses of intestinal morphology were executed by the same person.

### Statistical Analysis

Analysis of variance (**ANOVA**) was performed using the general linear model (GLM) procedure of SAS (9.1.3, 2003). The mean values were compared using Tukey's test. Polynomial contrasts were used to determine the linear and quadratic effects of DL-Met and L-Met levels on response criteria. Orthogonal contrasts were used for comparing L-Met and DL-Met within each supplementation level. The significance level was considered at *P* ≤ 0.05. The interactions between the 2 sources × 4 levels of Met were analyzed in a separate factorial arrangement of data to determine if an increasing dose of Met results in a different response for L compared with DL-Met.

To evaluate the RBA of the 2 Met sources, first, the response data were analyzed to verify the fundamental validity assumptions of the slope-ratio assay (Littell et al., 1997), which includes testing for linearity of the responses and equality of the common intercept to the mean of BD. Subsequently, the nonlinear exponential regression model was applied for estimation of RBA using the NLMIXED procedure of SAS (9.1.3, 2003) as follows:

$$y=a+b\times \left(1-\text{ex}p^{(c\times x_{1}}{{}^{+d\times x}}^{{}_{2})}\right)$$

Where, y = response (e.g., ADG, F:G ratio, etc.), a = intercept (the response achieved with BD), b = asymptotic response, a + b = common asymptote (the maximum performance), c = steepness coefficient for DL-Met, d = steepness coefficient for L-Met, and x_1_ and x_2_= dietary supplemental levels of DL-Met and L-Met, respectively. The RBA values (%) were obtained by the ratio of d/c.

## RESULTS

### Growth Performance

The overall ANOVA showed significant differences among the treatments in final body weight, ADG, ADFI, and F:G (*P* < 0.05, Table 3). In both experiments, Met supplementation elicited a linear response (*P* < 0.05) for ADG, F:G ratio, and final body weight, irrespective of the Met source. In both experiments, the orthogonal contrasts showed that Met-supplemented diets resulted in higher BW and ADG and better F:G compared with the BD (*P* < 0.05). The interactions of Met sources and levels were significant (*P* < 0.05) on final BW (Exp. 1, 2), ADG (Exp. 1), ADFI (Exp. 2), and F:G (Exp. 1, 2) indicating different response for L compared with DL-Met at different levels of Met supplementation. In Exp. 1, the broilers fed L-Met supplemented diets had higher BW and ADG and better F:G compared with those fed DL-Met supplemented diets (specifically at levels 0.8 and 1.6 g/kg), whereas in Exp. 2, only BW and F:G improved by L-Met supplementation (*P* < 0.05, orthogonal contrast L-Met vs. DL-Met specifically at level 1.6 g/kg, Table 3).

**Table 3:** Performance of broilers as affected by Met source and supplementation level.^1^

Met source	Supplemental level (g/kg)	Initial weight (g)		Final BW (g)		ADG^2^ (g)		ADFI^3^ (g)		F:G^4^	
		Exp. 1	Exp. 2	Exp. 1	Exp. 2	Exp. 1	Exp. 2	Exp.1	Exp. 2	Exp. 1	Exp.2
BD	0	41.56	211.20	193.4^e^	697.5^c^	15.18^e^	34.73^c^	29.56^a^	88.94abc	1.94^a^	2.56^a^
DL-Met	0.8	41.97	224.16	219.6^d^	729.8^b^	17.76^d^	36.11^bc^	29.49^bc^	91.46^a^	1.66^b^	2.53^a^
DL-Met	1.6	41.62	218.87	221.2^cd^	735.7^b^	17.96^cd^	36.91abc	29.22^bc^	89.83^ab^	1.62^bc^	2.43^a^
DL-Met	2.4	41.30	217.56	236.7^ab^	759.1^a^	19.54^ab^	38.68^ab^	28.31^bc^	79.80^e^	1.45^d^	2.06^c^
DL-Met	3.2	41.42	211.10	233.3abc	761.1^a^	19.19abc	39.28^a^	28.9^bc^	80.77^d^^e^	1.50^cd^	2.06^c^
L-Met	0.8	41.42	220.85	226.5bcd	734.5^b^	18.50bcd	36.69abc	28.12^bc^	86.33abcd	1.52bcd	2.35^ab^
L-Met	1.6	41.78	215.55	240.0^a^	764.5^a^	19.82^a^	39.21^a^	28.04^bc^	85.33bcd^e^	1.41^d^	2.17^bc^
L-Met	2.4	41.10	213.53	231.8abcd	760.5^a^	19.07abc	39.07^ab^	27.62^c^	81.39^d^^e^	1.44^d^	2.08^c^
L-Met	3.2	41.30	217.00	230.5abcd	760.4^a^	18.92abcd	38.81^ab^	28.30^bc^	83.48^cd^^e^	1.50^cd^	2.15^bc^
SEM	0.3	3.44	2.5	3.18	0.24	0.68	0.34	1.01	0.028	0.041	
*P*-values											
Overall		0.64	0.31	0.001	0.001	0.001	0.001	0.01	0.001	0.001	0.001
Linear (DL-Met)		-	-	0.001	0.001	0.001	0.001	0.12	0.001	0.001	0.001
Linear (L-Met)		-	-	0.001	0.001	0.001	0.004	0.23	0.001s	0.001	0.001
Quadratic (DL-Met)		-	-	0.001	0.015	0.001	0.738	0.61	0.012	0.001	0.265
Quadratic (L-Met)		-	-	0.001	0.001	0.001	0.033	0.08	0.171	0.001	0.005
Orthogonal contrasts											
BD*vs.* Met supplements		0.53	0.1	0.001	0.001	0.001	0.001	0.03	0.001	0.001	0.001
DL-Met*vs.* L-Met		0.39	0.77	0.041	0.002	0.025	0.17	0.001	0.16	0.001	0.034
0.8 DL-Met*vs.* 0.8 L-Met		0.21	0.57	0.1	0.31	0.050	0.6	0.01	0.004	0.002	0.016
1.6 DL-Met*vs.* 1.6 L-Met		0.66	0.74	0.001	0.001	0.001	0.01	0.03	0.016	0.001	0.001
2.4 DL-Met*vs.* 2.4 L-Met		0.54	0.72	0.21	0.44	0.23	0.65	0.21	0.43	0.96	0.86
3.2 DL-Met*vs.* 3.2 L-Met		0.81	0.56	0.48	0.45	0.49	0.54	0.31	0.12	0.89	0.14
Level		-	-	0.002	0.001	0.001	0.001	0.18	0.001	0.001	0.001
Met Source		-	-	0.03	0.01	0.02	0.15	0.002	0.15	0.001	0.025
Level × Met Source		-	-	0.001	0.008	0.001	0.24	0.73	0.007	0.004	0.003

1 Each value is the mean of six replicates per treatment (10 chicks per replicate).

2 ADG = Average daily gain.

3 ADFI = Average daily feed intake.

4 F:G = Feed to gain ratio. Exp. = Experiment.

a,b,c,d in each column, means with no common superscripts differ significantly (*P* < 0.05). SEM = Standard error of means.

**Figure 1:**
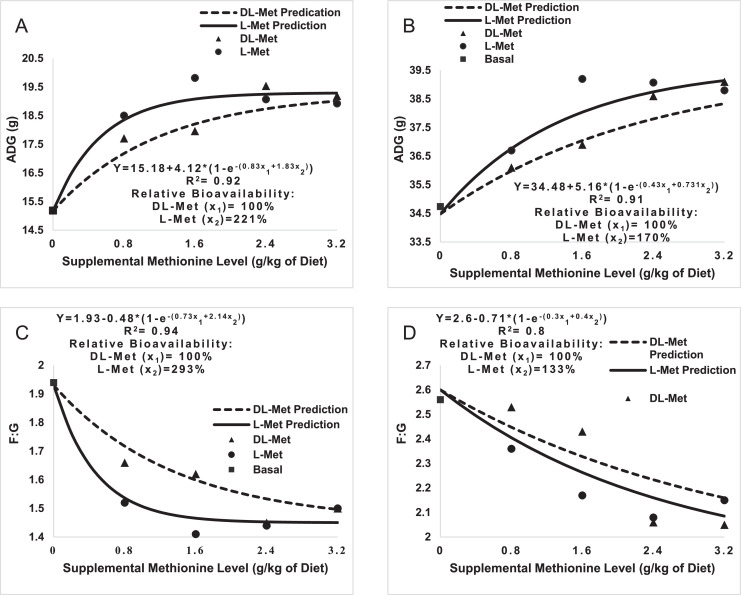
Relative bioavailability (RBA) of methionine sources in broilers, based on ADG and feed to gain ratio (F:G) by the nonlinear exponential model (A: ADG, 0–10 d; B: ADG, 11–24 d; C: F: G, 0–10 d; D: F: G, 11–24 d).

Figure 1 presents the RBA values of the Met sources based on performance criteria. The RBA of L-Met versus DL-Met based on the ADG response was 221 (Panel A) and 170% (Panel B) in the starter and grower periods, respectively. Relative bioavailability of L-Met based on F:G ratio was 293 (Panel C) and 133% (Panel D) in the starter period and grower period, respectively.

### Carcass Characteristics

**Figure 2:**
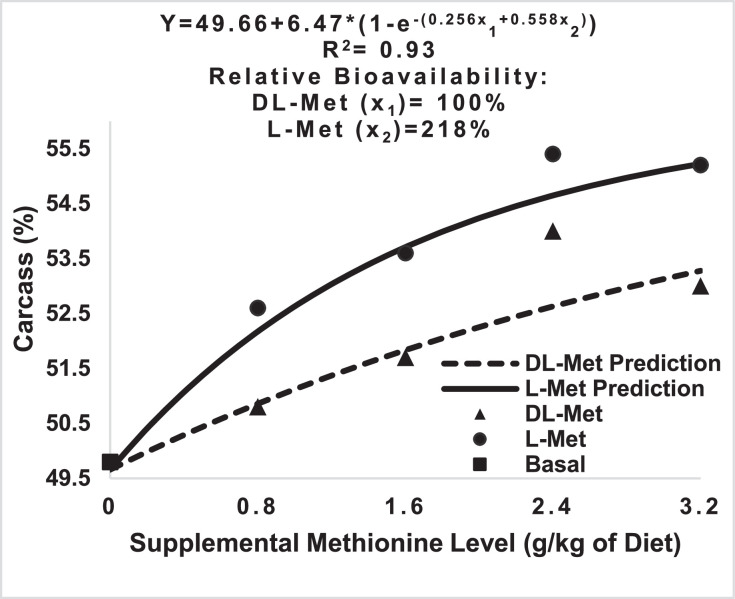
Relative bioavailability of methionine sources in broilers based on Carcass percentage (10 d) by the nonlinear exponential model.

In both experiments, supplementation of BD with Met sources significantly improved the relative weights of breast muscle and reduced the liver and abdominal fat weights (Table 4). In both experiments, L-Met linearly increased the relative weights of both carcass and breast muscle (*P* < 0.05), whereas DL-Met had a significant linear effect only on breast muscle percentage. Comparison of L-Met versus DL-Met showed significant differences in the carcass and breast percentages in the starter period (*P* < 0.05, Exp. 1) and relative liver weight in the grower phase (*P*<0.05, Exp. 2). No significant differences were observed in thymus, bursa of Fabricius, and spleen percentages (data not presented). Based on carcass weight response (218%), the RBA of L-Met was significantly higher than that of DL-Met only in the starter period (Figure 2).

**Table 4:** Carcass traits of broilers as affected by Met source and supplementation level.^1^

Met source	Supplemental level	Carcass (%)		Breast muscle (%)		Liver (%)		Abdominal fat (%)	
		Exp.1	Exp. 2	Exp.1	Exp.2	Exp.1	Exp.2	Exp. 1	Exp. 2
BD	0	49.81^c^	57.23	11.99^d^	18.80^b^	3.78^a^	2.99^a^	0.68^a^	1.35
DL-Met	0.8	50.89 cd	56.84	12.18^d^	19.71^ab^	3.19^b^	2.95^ab^	0.59 a	1.13
DL-Met	1.6	51.79 bcd	58.56	13.9^cd^	20.33^a^	3.18 b	2.74abc	0.56 ab	1.27
DL-Met	2.4	54.05 ab	58.47	15.06 bc	20.19^a^	3.24 b	2.63^bc^	0.55 ab	1.10
DL-Met	3.2	53.45 abc	57.60	16.04^ab^	20.12^a^	3.12 b	2.65^bc^	0.52 ab	1.02
L-Met	0.8	52.62 abcd	57.37	12.29^d^	19.30^ab^^b^	3.32 b	2.72abc	0.53^ab^	1.08
L-Met	1.6	53.60 abc	57.27	13.87 cd	20.03^a^	3.10 b	2.55^c^	0.53^ab^	0.97
L-Met	2.4	55.42 a	59.50	16.92 ab	20.45^a^	3.10 b	2.50 c	0.51^b^	1.06
L-Met	3.2	55.22 a	58.63	17.84^a^	20.08^a^	3.18 b	2.60 c	0.52^ab^	1.10
SEM		0.93	0.71	0.6	0.45	0.08	0.1	0.04	0.13
*P-*values									
Overall		0.001	0.055	0.001	0.02	0.001	0.001	0.02	0.27
Linear (DL-Met)		0.001	0.31	0.001	0.010	0.001	0.001	0.001	0.6
Linear (L-Met)		0.001	0.01	0.001	0.001	0.001	0.001	0.001	0.13
Quadratic (DL-Met)		0.26	0.48	0.63	0.11	0.03	0.58	0.65	0.8
Quadratic (L-Met)		0.04	0.37	0.14	0.14	0.001	0.005	0.001	0.06
Orthogonal contrasts									
BD *vs.* Met supplements		0.001	0.5	0.001	0.001	0.001	0.001	0.001	0.03
DL-Met *vs.* L-Met		0.001	0.5	0.006	0.81	0.96	0.005	0.29	0.31
0.8 DL-Met *vs.* 0.8 L-Met		0.15	0.06	0.87	0.65	0.07	0.026	0.17	0.79
1.6 DL-Met *vs.* 1.6 L-Met		0.05	0.26	0.92	0.62	0.84	0.06	0.29	0.06
2.4 DL-Met *vs.* 2.4 L-Met		0.14	0.26	0.004	0.48	0.13	0.19	0.34	0.74
3.2 DL-Met *vs.* 3.2 L-Met		0.05	0.96	0.005	0.88	0.63	0.69	0.25	0.47
Level		0.001	0.04	0.001	0.15	0.16	0.001	0.12	0.9
Met Source		0.001	0.47	0.004	0.63	0.89	0.004	0.07	0.22
Level × Met Source		0.98	0.17	0.04	0.77	0.14	0.59	0.08	0.17

1 Each value is the mean of twelve samples per treatment. Exp= Experiment.

a,b,c,d in each column, means with no common superscripts differ significantly (*P* < 0.05). SEM = Standard error of means.

### Serum and Antioxidant Status

Regardless of Met sources, supplementation with Met increased serum GSH-Px_2_ activity and reduced MDA concentration in both experiments (*P* < 0.05, Table 5). Broilers fed L-Met supplemented diets had lower serum MDA concentration in comparison with DL-Met in the Exp. 1 (*P* < 0.05, Table 5).

**Table 5:** Serum antioxidant status and biochemical indices of broilers as affected by Met source and supplementation level.^1^

Met source	Supplemental level	GSH-Px_2_^2^ (U/L)		MDA^3^ (nMol/mL)		Albumin (g/dL)		Total protein (g/dL)		Uric acid (mg/dL)	
		Exp. 1	Exp. 2	Exp. 1	Exp. 2	Exp. 1	Exp. 2	Exp. 1	Exp.2	Exp. 1	Exp.2
BD	0	848.6 ^e^	1038.2^b^	4.84^a^	4.08	2.44 b	3.68 b	4.38 b	4.73 b	6.08	6.29
DLM	0.8	1137.5^cd^^e^	1039^b^	4.32^ab^	3.82	2.59 ab	3.77^ab^	4.54^ab^	4.94^ab^	5.82	6.93
DLM	1.6	1115 cd^e^	1605^a^	4.03^ab^	3.95	2.60^ab^	3.81 ab	4.52 ab	5.62 ab	5.71	7.73
DLM	2.4	1481.4 ab	1637^a^	3.86 ab	3.59	2.62^a^	4.09^a^	4.55 ab	6.34 ab	5.89	7.6
DLM	3.2	1381.7abc	1090^b^	3.42 ab	3.98	2.66^a^	3.92 ab	4.71 ab	6.46 ab	5.88	7.48
LM	0.8	908.8 d^e^	1098^b^	3.45^ab^	3.64	2.56^ab^	3.89 ab	4.51 ab	5.14 ab	5.8	7.6
LM	1.6	1208 abcd	1388^ab^	3.32 b	3.6	2.66^a^	3.9 ab	4.72 ab	5.75 ab	5.92	7.34
LM	2.4	1509^a^	1620^a^	3.49 ab	3.55	2.69 a	4.12^a^	4.87^a^	6.18 ab	6	7
LM	3.2	1157.5bcd	1256 ab	3.22 b	3.88	2.62 a	4.01 ab	4.69 ab	7.24 a	5.98	7.09
SEM	83.5	94.6	0.39	0.37	0.049	0.01	0.12	0.58	0.4	0.69	
*P*-values											
Overall		0.001	0.001	0.006	0.08	0.001	0.004	0.01	0.04	0.71	0.65
Linear (DL-Met)		0.001	0.033	0.003	0.47	0.001	0.04	0.02	0.002	0.41	0.28
Linear (L-Met)		0.001	0.004	0.01	0.43	0.0003	0.001	0.003	0.001	0.93	0.61
Quadratic (DL-Met)		0.086	0.001	0.84	0.11	0.14	0.45	0.82	0.97	0.21	0.58
Quadratic (L-Met)		0.001	0.014	0.08	0.02	0.004	0.19	0.06	0.52	0.5	0.052
Orthogonal contrasts BD vs. Met supplements		0.001	0.003	0.001	0.047	0.001	0.005	0.006	0.03	0.26	0.09
DL-Met vs. L-Met		0.1	0.96	0.024	0.1	0.22	0.2	0.081	0.54	0.26	0.48
0.8 DL-Met *vs.* 0.8 L-Met		0.049	0.62	0.06	0.34	0.85	0.3	0.83	0.82	0.87	0.41
1.6 DL-Met *vs.* 1.6 L-Met		0.33	0.05	0.14	0.1	0.09	0.48	0.13	0.84	0.36	0.65
2.4 DL-Met *vs.* 2.4 L-Met		0.77	0.88	0.46	0.81	0.11	0.84	0.02	0.8	0.41	0.55
3.2 DL-Met *vs.* 3.2 L-Met		0.03	0.13	0.63	0.67	0.46	0.52	0.87	0.34	0.48	0.26
Level		0.001	0.001	0.28	0.9	0.15	0.006	0.2	0.03	0.57	0.87
Met Source		0.11	0.96	0.02	0.18	0.43	0.22	0.09	0.56	0.26	0.51
Level × Met Source		0.056	0.09	0.66	0.53	0.25	0.94	0.2	0.86	0.87	0.61

1 Each value is the mean of twelve samples per treatment.

2 GSH-Px2 = glutathione peroxidase.

3 MDA = malondialdehyde. Exp= Experiment.

a,b,c,d in each column, means with no common superscripts differ significantly (*P* < 0.05). SEM = Standard error of means.

In both experiments, supplementation with L-Methionine resulted in a linear increase (*P* < 0.05) in GSH-Px2 activity. However, DL-Methionine supplementation showed quadratic improvements in the activity of the enzyme in experiment 2. In the first experiment, both sources of Methionine led to a linear decrease in serum MDA concentration, whereas in the second experiment, only L-Methionine quadratically decreased serum MDA concentration.

Chicks fed diets supplemented with Met (both sources) had higher serum albumin and total protein concentrations in both experiments (*P* < 0.05, Table 5); however, serum uric acid levels were not affected by Met supplementation.

### Intestinal Villus Morphology

The histological parameters of the jejunum of the broilers are presented in Table 6. In Exp. 1 The crypt depth of chicks fed Met-supplemented diets was lower (*P* < 0.05) compared with that of chicks fed BD. The ratio of villus height to crypt depth was higher in the Met-supplemented group than that in the BD-supplemented group (*P* < 0.05). The experiments demonstrated that L-MET supplementation led to a significant quadratic increase in both villus height and the villus height to crypt depth ratio (*P* < 0.05); However, DL-Met supplementation was only effective in linear improvements of the villus height to crypt depth ratio in experiment 1.

**Table 6:** Jejunum morphology of broilers as affected by Met source and supplementation level.^1^

Met source	Supplemental level	Villus height (μm)		Crypt depth (μm)		Villus width (μm)		Villus height: crypt depth	
		Exp. 1	Exp. 2	Exp. 1	Exp. 2	Exp. 1	Exp. 2	Exp. 1	Exp. 2
BD	0	1290^b^	1485	230^a^	256	107	126	5.61^b^	5.80^c^
DLM	0.8	1370^ab^	1595	225^a^	210	99	110	6.08^bc^	7.59abc
DLM	1.6	1425^ab^	1565	225^a^	250	108	122	6.33 abc	6.26 bc
DLM	2.4	1418^ab^	1635	181.2^b^	202	108	125	7.82abc	8.09 ab
DLM	3.2	1600^a^	1715	195 ab	192	92	105	8.21 ab	8.93^a^
LM	0.8	1637^a^	1735	193.7^ab^	210	106	118	8.45^a^	8.26 a
LM	1.6	1490^ab^	1605	195^ab^	182	88	82	7.64abc	8.81 a
LM	2.4	1443^ab^	1515	193.7 ab	206	88	98	7.44abc	7.35 abc
LM	3.2	1392^ab^	1520	200 ab	210	97	94	6.96 abc	7.23 abc
SEM		61.3	76.95	10.54	16.37	8.5	17.9	0.95	0.68
*P*-values									
Overall		0.038	0.28	0.045	0.062	0.16	0.07	0.014	0.04
Linear (DL-Met)		0.07	0.053	0.01	0.03	0.13	0.47	0.01	0.06
Linear (L-Met)		0.91	0.44	0.03	0.01	0.01	0.008	0.12	0.38
Quadratic (DL-Met)		0.49	0.88	0.67	0.81	0.67	0.68	0.93	0.64
Quadratic (L-Met)		0.001	0.06	0.01	0.04	0.02	0.8	0.001	0.02
Orthogonal contrasts BD vs. Met supplements		0.037	0.13	0.007	0.01	0.02	0.10	0.003	0.01
DL-Met vs. L-Met		0.44	0.53	0.19	0.34	0.11	0.02	0.25	0.63
0.8 DL-Met *vs.* 0.8 L-Met		0.01	0.2	0.1	1	0.79	0.6	0.01	0.44
1.6 DL-Met *vs.* 1.6 L-Met		0.45	0.71	0.06	0.007	0.1	0.01	0.08	0.01
2.4 DL-Met *vs.* 2.4 L-Met		0.83	0.27	0.49	0.87	0.1	0.08	0.51	0.37
3.2 DL-Met *vs.* 3.2 L-Met		0.02	0.08	0.74	0.46	0.67	0.5	0.08	0.15
Level		0.68	0.63	0.17	0.75	0.69	0.49	0.63	0.74
Met Source		0.42	0.53	0.17	0.28	0.16	0.035	0.23	0.63
Level × Met Source		0.01	0.13	0.11	0.03	0.08	0.18	0.007	0.03

1 Each value is the mean of twelve samples per treatment. Exp= Experiment.

a,b,c,d in each column, means with no common superscripts differ significantly (*P* < 0.05). SEM = Standard error of means.

## DISCUSSION

In both experiments, Met supplementation improved the growth performance. This result is consistent with previous studies (Shen et al., 2015; Conde-Aguilera et al., 2016; Zhang et al., 2019). Methionine supplementation improves performance through several mechanisms. Met is one of the critical amino acids for protein synthesis. Del Vesco et al. (2015) found that dietary Met supplementation positively affects the expression of genes related to growth and protein synthesis, including insulin-like growth factor I (**IGF-I**) and hepatic growth hormone receptor (**GHR**), and decreases the expression of genes that participate in protein degradation, such as atrogin 1 and cathepsin L2, in the breast muscle of broilers. Dietary deficiency of Met leads to lower performance, poor uniformity, and increased fat deposition (Vieira et al., 2004; Corzo et al., 2006). Similarly, inadequate dietary Met has negative impacts on body weight, feed intake, and feed efficiency in turkey poults (Gonzales-Esquerra et al., 2007).

The chicks fed L-Met supplemented diets (specifically at levels 0.8 and 1.6 g/kg) showed higher final BW, lower F:G in both experiments, and better ADG in Exp. 1 compared with chicks fed DL-Met supplemented diets. Moreover, based on carcass response, the RBA of L-Met compared to DL-Met was higher in the starter phase (218%). These findings are consistent with recent studies on broiler chickens (Shen et al., 2015; Millecam et al., 2021), turkeys (Park et al., 2018) and Pekin ducks (Zhang et al., 2019). These data indicate that L-Met had better bioavailability than DL-Met because L-Met can be used directly in protein synthesis, whereas part of DL-Met requires deamination to keto-methionine and then transamination to L-Met before it can be incorporated into protein (Dilger et al., 2007). Conversion of D-Met to L-Met may limit broiler performance, especially during the starter period. According to D'Aniello et al. (1993), younger animals have lower amounts of D-amino-acid oxidase and consequently, higher concentrations of free D-amino-acid compared to adult animals. Similarly, Zhang et al. (2019) reported a higher RBA for L-Met than DL-Met in Pekin ducks in the starter phase but not in the grower phase. Our results further indicated that the highest performance in each experiment (Table 3) was achieved by lower levels of L-Met supplementation (1.6–2.4 g/kg) than DL-Met supplementation (2.4–3.2 g/kg). Millecam et al. (2021) re-evaluated the optimal sulfur amino acid (SAA) requirements of broilers using L-Met supplementation and concluded that compared to conventional DL-Met supplementation, broilers have lower SAA requirements based on L-Met supplementation; however, further research is required to confirm these findings.

The RBA value of L-Met compared to DL-Met based on ADG response in the starter phase (221%) was comparable with the finding of Shen et al. (2015) in the starter phase of broiler chicks (217%) but higher than the values (141.5 %) reported by Esteve-Garcia and Khan (2018) and Wang et al. (2019) for broiler chickens (141.5%), Park et al. (2018) for starting turkey poults (160%), and Zhang et al. (2019) for the starter phase of Pekin ducks (138 %). It should be noted that the resultant RBA value was not considered significant in this experiment due to the overlap of the 95% confidence interval of the bioavailability (59–383%) with 100% (DL-Met). Except Wang et al. (2019), the aforementioned researchers did not consider this fact in their reports. The RBA value of L-Met vs. DL-Met based on F:G response in the starter phase (293%) was lower than the value obtained by Shen et al. (2015) in the starter phase of broiler chicks (437%); however, it was higher than the values reported by Wang et al. (2019) and Zhang et al. (2019) for the starter phase of Pekin ducks (121%). The RBA estimate based on the F:G response was not considered significant because the 95% confidence interval (88–497%) overlapped with 100% (DL-Met). No experiments were found to be conducted separately in the grower phase of broiler chicks regarding the bioavailability of L-Met. The RBA values of L-Met versus DL-Met in the grower phase (11–24 d of age, 170 and 133% based on ADG and FCR, respectively) are consistent with those reported by Shen et al. (2015) from d 1 to d 21 (138 and 141% based on ADG and FCR, respectively). Differences in the bioavailability values in different experiments can be related to animal species, strain, age, statistical methods, and Met source.

In the present study, BD supplementation with either L-Met or DL-Met improved the carcass and breast muscle yield and reduced the liver and abdominal fat content. These findings are consistent with previous studies (Waldroup et al., 2006; Daskiran et al., 2009). Dietary Met deficiency increases body protein breakdown (Tesseraud et al., 2007) and reduces protein synthesis (Kimball and Jefferson, 2006). Met deficiency has been shown to affect the chemical composition of different tissues and certain aspects of breast meat quality of broilers (Conde-Aguilera et al., 2013). It has been speculated that a marginal Met deficiency is compensated by additional feed intake, leading to an increase in body fat (Esteve-Garcia and Llaurado, 1997). Fat may accumulate in the liver when the dietary Met level is low (Del Vesco et al., 2013), which might explain the higher liver weight of the chickens fed BD in the present study. The RBA of L-Met compared to DL-Met based on carcass traits was higher in the starter phase, but no significant differences were observed in the grower phase.

Met supplementation of BD significantly increased GSH-Px_2_ activity in both experiments and reduced serum MDA concentration in Exp. 1 (Table 5). This finding agrees with the result of Park et al. (2018), who observed that Met supplementation tended to decrease MDA and increase glutathione in the liver of turkey poults compared with a basal diet without Met supplementation. Similarly, Shen et al. (2015) demonstrated that supplementation with either L-Met or DL-Met has beneficial effects on villus development due to increased glutathione production and reduced protein oxidation in the duodenum. Met is a precursor of other sulfur-containing amino acids (L-cysteine and taurine) involved in the antioxidant system (Mukwevho et al., 2014). Glutathione indirectly regulates epithelial cell proliferation via modulation of redox status (Shoveller et al., 2003). Met stimulates glutathione redox systems, including the activity of glutathione peroxidase, glutathione reductase, and glutathione transferase (N´emeth et al., 2004; Blaszczyk et al., 2009).

Supplementation of BD with Met resulted in higher serum albumin and total protein levels, in agreement with previous studies (Hern´andez et al., 2012; Ospina-Rojas et al., 2014). Serum uric acid concentrations did not change with Met supplementation in both experiments. Similar studies in broilers (Wang et al., 2019) and pigs (Shen et al., 2014) found that uric acid levels decreased with increasing Met levels, of which L-Met was more effective.

Our results suggest that chicks fed diets supplemented with either L- or DL-Met had higher villus height:crypt depth ratio in the first experiment and lower crypt depth compared with chicks fed BD. Met plays a positive role in villus growth and maintains small intestine integrity (Shen et al., 2015; Yang and Liao, 2019). Improvement in the intestinal histo-morphometric parameters indicates improved intestinal maturity, and thus, higher absorption of nutrients (Hou and Tako, 2018). The gastrointestinal tract has a functional requirement for Met (Shoveller et al., 2003); thus, the availability of this critical nutrient can elevate the efficiency of absorption and, consequently, improve the production traits.

## CONCLUSIONS

Synthetic Met (L- or DL-) supplementation of the BD has beneficial effects on broiler production traits. Orthogonal contrasts revealed that broiler chicks supplemented with L-Met specifically at the level of 1.6 g/kg, compared to those supplemented with DL-Met, exhibited higher growth performance, carcass traits, and antioxidant status, particularly during the starter phase. Furthermore, the RBA of L-Met relative to DL-Met based on carcass percentage was significantly higher in the starter phase; however, this advantage disappeared in the grower phase. Therefore, further investigation into the RBA of L-Met as a new source of Met across different growth phases is warranted.

## DISCLOSURES

The authors declare no conflicts of interest.
